# HO-1197 as a Multifaceted Therapeutic: Targeting the Cell Cycle, Angiogenesis, Metastasis, and Tumor Immunity in Hepatocellular Carcinoma

**DOI:** 10.3390/ijms262110329

**Published:** 2025-10-23

**Authors:** Yeonhwa Song, Seungeun Lee, So-Won Heo, Juliane Spohn, Dominik Schmiedel, Taemoo Heo, Sanghwa Kim, Jongmin Park, Haeng Ran Seo

**Affiliations:** 1 Advanced Biomedical Research Lab, Institut Pasteur Korea, 16, Daewangpangyo-ro 712 beon-gil, Bundang-gu, Seongnam-si 13488, Gyeonggi-do, Republic of Korea; yeonhwa.song@ip-korea.org (Y.S.); seungeun.lee@ip-korea.org (S.L.); sowon.heo@ip-korea.org (S.-W.H.); taemoo.heo@ip-korea.org (T.H.); sanghwa.kim@ip-korea.org (S.K.); 2 Department of Advanced Drug Discovery & Development, Institut Pasteur Korea (IPK) School, University of Science and Technology (UST), 16, Daewangpangyo-ro 712 Beon-gil, Bundang-gu, Seongnam-si 13488, Gyeonggi-do, Republic of Korea; 3 Department for Bio- and Nanotechnology, Fraunhofer Institute for Ceramic Technologies and Systems (IKTS), Perlickstr. 1, 04103 Leipzig, Germany; juliane.spohn@ikts.frauhofer.de; 4 Department for Cell and Gene Therapy Development, Fraunhofer Institute for Cell Therapy and Immunology (IZI), Perlickstr. 1, 04103 Leipzig, Germany; dominik.schmiedel@izi.fraunhofer.de; 5 H&O Biosis Co., Ltd., 19-10, Jeongnamsandan-ro, Jeongnam-myeon, Hwaseong-si 18514, Gyeonggi-do, Republic of Korea; jmpark@hnobiosis.com

**Keywords:** hepatocellular carcinoma (HCC), cell cycle arrest, FOXM1, angiogenesis, metastasis, immune modulation

## Abstract

Hepatocellular carcinoma (HCC) is the most prevalent primary malignancy of the liver. Characterized by rapid progression and poor overall survival rates, HCC requires effective and streamlined treatment regimens. It predominantly occurs in East Asia and sub-Saharan Africa, where it has historically been managed with herbal formulas. We previously observed that the herbal formula HO-1089 exerts potent anti-HCC effects both in vitro and in vivo. In this study, we investigated the anticancer efficacy and mechanisms of HO-1197, a reconstituted herbal formulation derived from HO-1089. HO-1197 selectively inhibited the viability of HCC cell lines without hepatotoxicity and demonstrated superior anticancer activity compared with both HO-1089 and sorafenib. Mechanistically, HO-1197 induced apoptosis and G2/M arrest through reactive oxygen species-mediated DNA damage, independent of p53 status. Transcriptomic analysis revealed downregulation of mitosis-related genes, particularly those regulated by FOXM1, a key driver of HCC proliferation and metastasis. HO-1197 suppressed FOXM1 expression and nuclear translocation, reducing its downstream targets and diminishing angiogenic and metastatic potential. Furthermore, HO-1197 modulated the tumor immune microenvironment by promoting pro-inflammatory macrophage polarization and enhancing natural killer cell-mediated cytotoxicity. HO-1197 exhibited potent antitumor efficacy, and combination therapy with HO-1197 and sorafenib exhibited synergistic effects in both two-dimensional and immune-activated multicellular spheroid models. These findings suggest that HO-1197 is a promising multifunctional therapeutic candidate with antitumor, antiangiogenic, antimetastatic, and immunomodulatory properties. Its combination with sorafenib may offer effective treatment for HCC. HO-1197, which demonstrated strong efficacy, is a novel herbal medicine developed by H&O Biosis and is referred to as an Integrated Natural Medicine.

## 1. Introduction

Liver cancer is the third most common cause of cancer deaths worldwide [1]. Liver cancer generally has an unfavorable prognosis, even when diagnosed in its early stages [2]. The most common form of liver cancer is hepatocellular carcinoma (HCC), which accounts for 80–90% of liver cancer cases [3]. However, the two drug classes used for HCC therapy, namely, angiogenesis inhibitors and immune checkpoint inhibitors [4], do not significantly improve HCC outcomes [5].

Recent studies have shown that combining bevacizumab with atezolizumab increases overall survival and prolongs progression-free survival compared with sorafenib in patients with HCC [3]. This first-in-class combination of an immuno-oncology agent with an angiogenesis inhibitor represents a shift in strategy for drug discovery for HCC from monotherapies to combination regimens [6,7]. Herbal medicine is a classical form of combination therapy that has been empirically proven to retard HCC progression and prevent HCC occurrence [8,9,10]. However, the complex compositions of herbal formulas have hindered the elucidation of their mechanisms of action against HCC and the modernization of herbal medicine. Well-designed systematic clinical trials have recently been initiated to study the efficacy of herbal formulas in the treatment of HCC and to explore their underlying antitumor mechanisms in detail [11,12].

We previously reported that the herbal formula HO-1089 has anti-HCC effects in vivo and in vitro by inhibiting the expression of the G2/M phase regulatory protein polo-like kinase 1 (PLK1) [13]. To develop a formula with greater anticancer efficacy, we generated HO-1197 by recombining the five herbs of HO-1089: *Herniariae herba*, *A. fructus*, prunella spike, *C. zedoaria* Rosc. and Curcuma root. These plants contain a wide range of phytoconstituents and have diverse biological activities. Curcuma root contains 89 curcuminoids, including three major curcuminoids: curcumin, demethoxycurcumin, and bismethoxycurcumin [14]. The medicinal components of *A. fructus* are bornyl acetate and borneol [15]. *H. herba* mainly contains saponins, flavonoids, rutin, quercetin, and H. herba treatment caused lowering of cell viability in various human cancer cell lines [16]. The constituents of the prunella spike include ursolic acid, oleanolic acid, prunellin, rutin, and hyperoside [17]. *C. zedoaria* Rosc. has a complex range of phytoconstituents, including curcumin, ethyl p-methoxycinnamate, β-turmerone, β-eudesmol, zingiberene, dihydrocurcumin, furanodiene, α-phellandrene, 1,8-cineole, β-elemene, and germacrone [18]. Although anticancer effects of the various phytoconstituents of these five herbs have been reported, the anticancer efficacy of this assembly of ingredients against HCC has not been studied.

In this study, we treated HCC cells with HO-1197 and used data from RNA sequencing (RNA-seq) to perform a comprehensive transcriptomic analysis. We found that the cell cycle pathway is significantly associated with the inhibitory effects of HO-1197. We determined that HO-1197 dramatically inhibits the growth of HCC cells by altering the transcriptional regulation of G2/M regulatory proteins and perturbing the G2/M transition. HO-1197 has been designated as an Integrated Natural Medicine to reflect its multi-targeted and scientifically grounded therapeutic approach.

## 2. Results

### 2.1. HO-1197 Exhibits Selective Antitumor Activity Against HCC Cells Without Inducing Hepatocyte Toxicity

**Figure 1:**
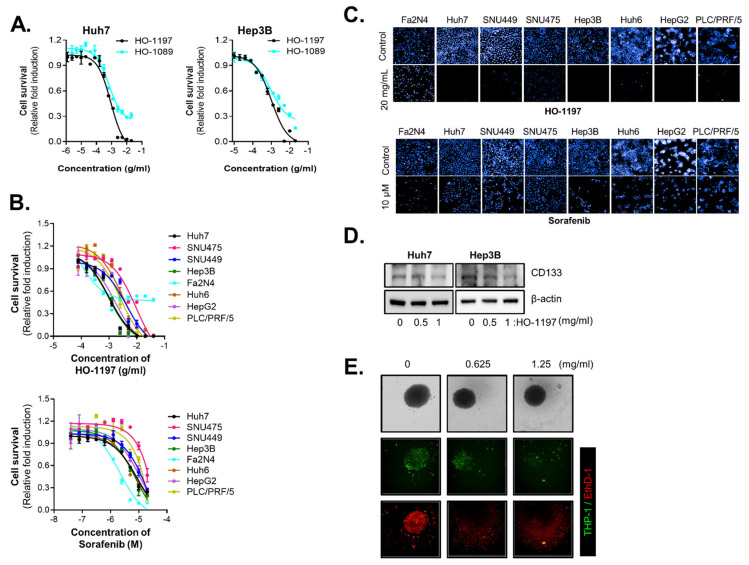
Antitumor effect of HO-1197 in HCC cells. (**A**) Huh7 and Hep3B cells were treated with HO-1089 (starting at 40 mg/mL) or HO-1197 (starting at 40 mg/mL) for 48 h. Cell survival was assessed via nuclear staining analysis. (**B**) Normal hepatocytes (Fa2N-4) and hepatocellular carcinoma (HCC) cells (Huh7, Hep3B, SNU475, SNU449, Huh6, HepG2, and PLC/PRF/5) were treated with 2-fold sequential serial dilutions of HO-1197 (beginning at 40 mg/mL) and sorafenib (beginning at 20 μM) for 48 h. Cell viability was measured by counting the number of cell nuclei. (**C**) Morphology and number of nuclei after treatment with 20 mg/mL HO-1197 or 10 μM sorafenib for 48 h. (**D**) CD133 protein expression was measured in Huh7 and Hep3B cells treated with HO-1089 or HO-1197 (0, 0.5, or 1 mg/mL). (**E**) Immune-activated multicellular HCC spheroids were treated with HO-1197 (0, 0.625, or 1.25 mg/mL) for 72 h. Fluorescence staining was performed for macrophages (THP-1), and EthD-1 was used to detect cell death. All images were obtained using the HCS system, and values were calculated using Harmony software (version 5.1).

In our previous study, we demonstrated the therapeutic efficacy of the traditional herbal formulation HO-1089 in HCC through both in vitro and in vivo models. To evaluate the antitumor efficacy of HO-1197, which is a reconstituted herbal formulation selected from components of HO-1089, we compared its inhibitory effects on HCC cell proliferation with those of the original formulation, HO-1089. HO-1197 demonstrated enhanced cancer cell cytotoxicity compared with higher concentrations of HO-1089, suggesting that the reconstitution of key bioactive compounds potentiates its anticancer efficacy [Figure 1A]. The results of the dose–response curve analysis demonstrated that HO-1197 was a more potent inhibitor of HCC cell lines such as Huh7 (IC50 1 mg/mL), Hep3B (IC50 1 mg/mL), Huh6 (IC50 3 mg/mL), HepG2 (IC50 1.7 mg/mL), PLC/PRF/5 (IC50 2.5 mg/mL), SNU449 (IC50 4.3 mg/mL), and SNU475 (IC50 9.5 mg/mL) than of normal hepatocytes (Fa2N-4). Also, LX2 and WI38 exhibited similar cytotoxicity with cancer cell lines [Supplementary Figure S1]. By contrast, sorafenib had greater cytotoxic effects on normal hepatocytes than on HCC cells [Figure 1B]. To confirm the selectivity of HO-1197 for HCC cells, we observed images of the nuclei of HCC cells treated with 20 mg/mL HO-1197. Compared with treatment with 10 µM sorafenib, treatment with HO-1197 significantly reduced the number of nuclei in various HCC cells but not in normal hepatocytes [Figure 1C]. Single- and repeated-dose toxicity studies of HO-1197 in rats showed that HO-1197 is safe and nontoxic (Corestemchemon, study number:23-RA-0274N). In our previous studies, we observed that treatment with high concentrations of HO-1089 [13] and sorafenib [19] did not effectively eliminate CD133+ cancer stem cells in HCC. However, HO-1197 treatment led to complete eradication of various HCC cell populations. To evaluate the cancer stem cell eradication potential of HO-1197 in HCC, we detected CD133 expression following HO-1197 treatment and found that it was significantly reduced in response to this treatment [Figure 1D].

To further assess the effectiveness of HO-1197 in a more physiologically relevant setting, we employed a multicellular HCC spheroid model incorporating macrophages to mimic the complex tumor microenvironment (TME). HO-1197 treatment significantly reduced the overall size of the multicellular HCC spheroids, demonstrating its ability to suppress tumor growth in an immune cell-enriched microenvironment [Figure 1E]. These findings suggest that HO-1197 not only has greater anticancer effects than HO-1089 without damaging hepatocytes but also exerts potent antitumor effects within a more clinically relevant tumor context.

### 2.2. HO-1197 Induces DNA Damage via ROS Accumulation in a p53-Independent Manner

**Figure 2:**
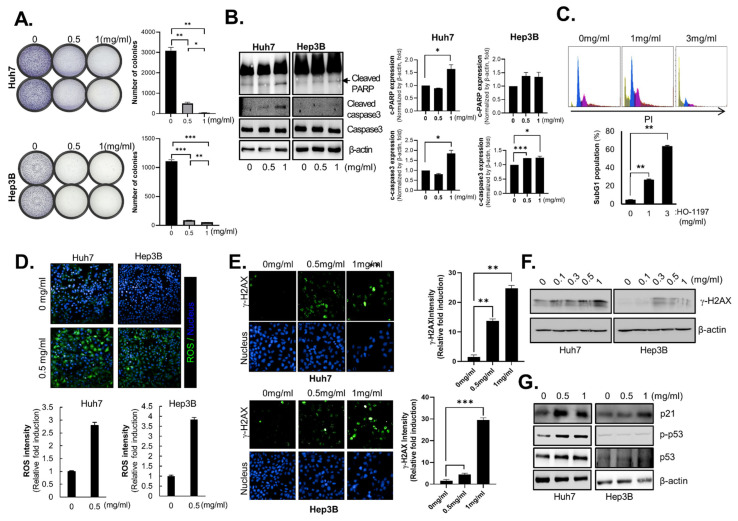
HO-1197 treatment induces ROS accumulation and subsequent DNA damage in a p53-independent manner. (**A**) Images of the colony formation assays of Huh7 and Hep3B cells treated with 0.5 or 1 mg/mL HO-1197 (**left**) and number of colonies calculated using AID vSpot Spectrum (**right**). (**B**) Immunoblotting of cleaved caspase-3 and cleaved PARP, indicators of apoptosis, in Huh7 and Hep3B cells treated with 0, 0.5, or 1 mg/mL HO-1197 for 48 h. (**C**) The percentage of sub-G1 populations in Huh7 cells treated with 0, 1, or 3 mg/mL HO-1197 for 48 h was determined by flow cytometry. (**D**) Huh7 and Hep3B cells were treated with 0.5 mg/mL HO-1197 for 24 h. ROS were detected by CMH2DCFDA staining and analysis using the HCS system (**up**). ROS intensity was measured and calculated (**down**). (**E**) The DNA damage-related protein γ-H2AXwas detected by imaging in Huh7 and Hep3B cells treated with 0, 0.5, or 1 mg/mL HO-1197 (**left**), and γ-H2AX intensity was calculated using the HCS system (**right**). (**F**) γ-H2AX protein expression in Huh7 and Hep3B cells treated with 0, 0.1, 0.3, 0.5, or 1 mg/mL HO-1197. (**G**) Protein expression of p21, p53, or p-p53 in Huh7 and Hep3B cells treated with 0, 0.5, or 1 mg/mL HO-1197 was measured by immunoblotting. All images were obtained using the HCS system, and values were calculated with Harmony software. The results were normalized to the control values to obtain the relative fold induction. Data are expressed as the mean ± SD (n = 3). * *p* < 0.05, ** *p* < 0.005, *** *p* < 0.0005 compared with the control group.

Treatment of Huh7 and Hep3B cells with HO-1197 significantly reduced clonogenic survival [Figure 2A]. To determine whether the inhibition of HCC cell growth by HO-1197 was related to an increase in apoptosis, we analyzed markers of apoptosis. The expression of cleaved PARP and cleaved caspase-3 increased in Huh7 and Hep3B cells treated with HO-1197 [Figure 2B]. Flow cytometry analysis showed that HO-1197 treatment increased the sub-G1 peak in a dose-dependent manner [Figure 2C]. We previously reported that HO-1089 induces DNA damage in HCC cells Via ROS accumulation. We therefore examined whether HO-1197 induces ROS accumulation in HCC cells. Treatment of Huh7 and Hep3B cells with 0.5 mg/mL HO-1197 significantly increased ROS production [Figure 2D], but not in LX2 and WI38 cells [Supplementary Figure S2]. Because excessive ROS accumulation can cause DNA damage and cell death, the DNA damage response was examined in HCC cells treated with HO-1197. HO-1197 treatment not only induced γ-H2AX foci formation [Figure 2E], a marker of DNA damage, but also increased γ-H2AX expression [Figure 2F] in a dose-dependent manner in HCC cells. However, both LX2 and WI38 exhibited markedly lower ROS induction compared to HCC cells [Supplementary Figure S3]. The ROS-induced DNA damage caused by HO-1197 treatment not only increased p21cip1/waf1 expression by activating p53 in Huh7 cells, which express wild-type p53, but also elevated p21cip1/waf1 expression in a p53-independent manner in Hep3B cells, which are p53 deficient [Figure 2G]. These results demonstrate that HO-1197 exerts anticancer effects by inducing DNA damage and apoptosis through excessive ROS accumulation in HCC cells.

### 2.3. HO-1197 Suppresses FOXM1 Activity by Inhibiting Its Expression and Nuclear Localization in the HCC Microenvironment

**Figure 3:**
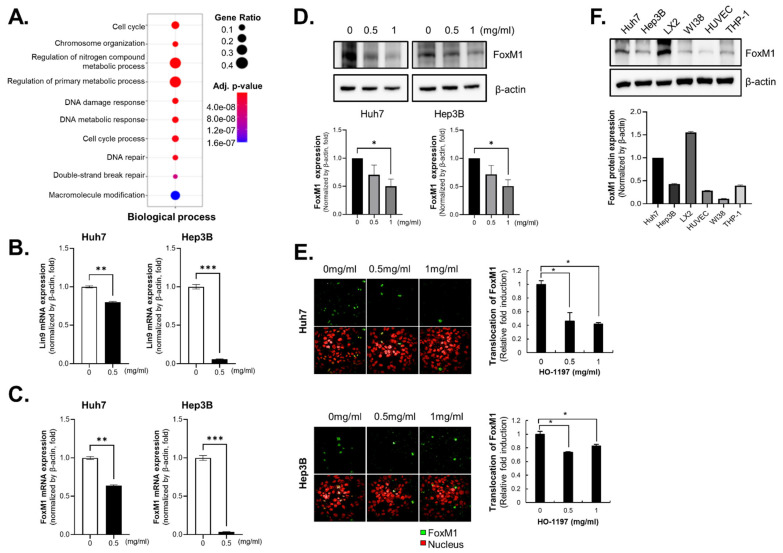
HO-1197 attenuates FOXM1 by downregulating its expression and nuclear localization. (**A**) The biological pathway of HO-1197 was analyzed by RNA sequencing of Huh7 cells after treatment with 1 mg/mL HO-1197 for 48 h. (**B**,**C**) The mRNA expression of Lin9 (**B**) and FoxM1 (**C**) was examined by qRT-PCR in Huh7 and Hep3B treated with HO-1197 (0 or 0.5 mg/mL) for 48 h. The results were normalized to the expression of β-actin to calculate relative fold induction. (**D**) FoxM1 protein expression was measured in Huh7 and Hep3B cells treated with 0, 0.5, or 1 mg/mL HO-1197. (**E**) The nuclear translocation of FoxM1 in Huh7 (**up**) and Hep3B (**down**) cells treated with 0, 0.5, or 1 mg/mL HO-1197 was examined by immunofluorescence staining (**left**) and calculated (**right**). (**F**) FoxM1 protein expression was detected in HCC cells (Huh7 and Hep3B), hepatic stellate cells (LX2), fibroblasts (WI38), epithelial cells (HUVECs), and macrophages (THP-1), which constitute tumor spheroids. All images were obtained using the HCS system, and values were calculated with Harmony software. Data are expressed as the mean ± SD (n = 3). * *p* < 0.05, ** *p* < 0.005, *** *p* < 0.0005 compared with the control group.

To determine the mechanism underlying the HCC cell–specific effects of HO-1197, we detected genome-wide alterations in Huh7 cells treated with HO-1197 by RNA-seq, a powerful approach for investigating drug-induced changes in transcription. Using a fold-change cutoff of > 3, we identified 762 differentially expressed genes in Huh7 cells treated with HO-1197. Of these 762 genes, the expression of 569 genes decreased in Huh7 cells treated with HO-1089. In terms of biological processes, the genes that were downregulated in HO-1197-treated cells were mainly related to cell cycle regulation, chromosome organization, DNA damage response, and DNA repair [Figure 3A]. The results of pathway analysis indicated that HO-1197 particularly altered the expression of mitosis-related proteins involved in all phases of the cell cycle [Supplementary Figure S4].

Because HO-1197 treatment altered the expression of a lot of mitosis-related proteins in HCC cells, we closely examined changes in the expression of transcription factors that regulate the cell cycle. RNA-seq analysis showed that lin-9 DREAM MuvB core complex component (Lin9), forkhead box M1 (FOXM1), and nuclear transcription factor Y subunit beta (NF-YB) expression were reduced by 7.002-, 7.36- and 4.27-fold, respectively, in HCC cells treated with HO-1197 compared with control cells. In Huh7 and Hep3B cells, HO-1197 treatment significantly reduced Lin9 and FOXM1 mRNA expression [Figure 3B,C] but not NF-YA and NF-YB mRNA expression [Supplementary Figure S5C,D].

FoxM1 and Lin9 are transcription factors that function during the S and G2/M phases of the cell cycle. Protein expression of FoxM1 decreased following HO-1197 treatment, whereas no significant change was observed after HO-1089 treatment in Huh7 and Hep3B cells [Figure 3D, Supplementary Figure S6]. Also, FoxM1 translocation in the cells from cytoplasm to nucleus was decreased by HO-1197 treatment in Huh7 and Hep3B cells [Figure 3E]. Because target genes of FoxM1 collectively promote tumor growth, metastasis, therapy resistance, and immune suppression, we analyzed FoxM1 expression in various cell types comprising the HCC microenvironment. FoxM1 was broadly expressed not only in HCC cells but also in stromal cells [Figure 3F]. FoxM1 protein stability was not affected by HO-1197 treatment, as shown by cycloheximide chase assay in Huh7 and Hep3B cells [Supplementary Figure S7].

### 2.4. HO-1197 Induces G2/M Cell Cycle Arrest by Downregulating Mitosis-Related Proteins in HCC Cells

**Figure 4:**
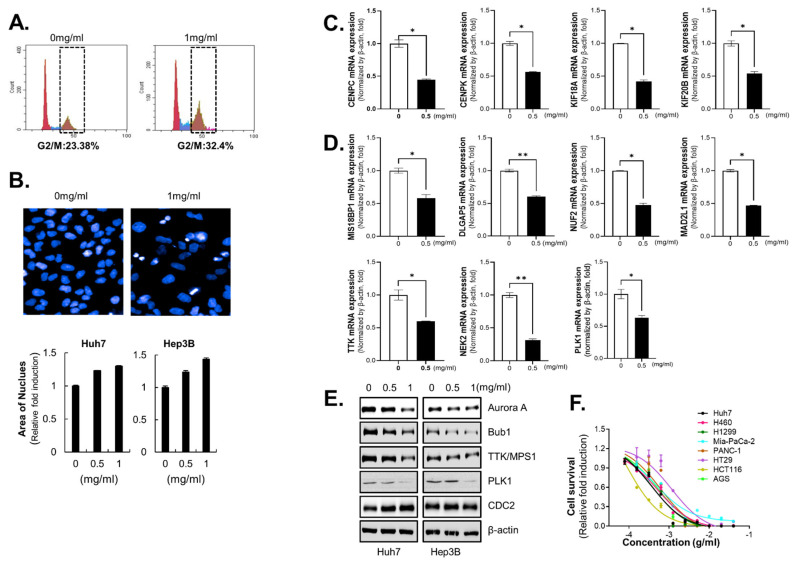
HO-1197 induces G2/M arrest in HCC cells by inhibiting the expression of mitosis-related proteins. (**A**) The percentage of the G2/M population in Huh7 cells treated for 48 h with 0 or 1 mg/mL HO-1197 was determined by flow cytometry. (**B**) The nuclear area in Huh7 and Hep3B cells treated with 0, 0.5, or 1 mg/mL HO-1197 was examined (Huh7, up) and calculated (down). (**C**,**D**) The expression of target genes in Huh7 or Hep3B cells treated with 0.5 mg/mL HO-1197 was examined by qRT–PCR. The expression of molecular motors (**C**) and spindle-assembly checkpoint (SAC)-related genes (**D**) was calculated after normalization to the expression of β-actin. The results were normalized to control values to obtain the relative fold induction. (**E**) The expression of mitosis-related genes was detected in Huh7 and Hep3B cells treated with 0, 0.5, or 1 mg/mL HO-1197. (**F**) HCC cells (Huh7), lung cancer cells (H460 and H1299), pancreatic cancer cells (Mia-PaCa-2 and PANC-1), colon cancer cells (HT29 and HCT116), and gastric cancer cells (AGS) were treated for 48 h with 2-fold sequential serial dilutions of HO-1197 (beginning at 40 mg/mL). Cell viability was measured by the number of cell nuclei. All images were obtained using the HCS system and analyzed using Harmony software. Data are expressed as the mean ± SD (n = 3). * *p* < 0.05 and ** *p* < 0.005 compared with the control group.

FoxM1 is a master regulator of cell cycle progression, particularly the G2/M transition (Via Plk1 and Cyclin B1) and mitotic execution (Via CENP-A and Survivin) [20]. As expected, cell cycle analysis revealed that the proportion of cells in G2/M arrest was greater in HO-1197-treated Huh7 cells than in control cells (32.4% versus 23.38%) [Figure 4A]. Cells in G2 phase have doubled their DNA content, which is often associated with enlarged nuclei. Thus, an increase in average nuclear size can serve as an indicator of cell cycle arrest at the G2/M transition. HCC cells treated with HO-1197 exhibited both apoptotic and enlarged nuclei [Figure 4B].

The genes that were significantly downregulated by HO-1197 included genes encoding proteins that control the attachment of mitotic spindle microtubules to the kinetochore during mitosis, such as centromere proteins, members of the kinesin family, and mitosis-related kinases. The changes in the expression of selected differentially expressed genes were analyzed by RT–PCR. The mRNA expression of CENPC, CENPK, KIF18A, and KIF20B, which encode molecular motors, was markedly decreased by HO-1197 treatment in Huh7 and Hep3B cells [Figure 4C, Supplementary Figure S5A]. HO-1197 treatment also suppressed the expression of spindle-assembly checkpoint (SAC)-related genes, including MIS18BP1, DLGA5, NUF2, MAD2L1, NEK2, and TTK, in HCC cells [Figure 4D, Supplementary Figure S5B]. Because HO-1197 mainly altered the mRNA expression of mitosis-related genes in HCC cells, we investigated the protein expression of major mitotic kinases: cyclin-dependent kinase 1 (CDK1/CDC2), budding uninhibited by benzimidazole 1 (BUB1), polo-like kinase 1 (PLK1), aurora kinase A (Aurora A), and monopolar spindle kinase 1 (MPS1/TTK) which are transcriptional targets of FoxM1. Interestingly, HO-1197 treatment sufficiently decreased Aurora A, BUB1, MPS1/TTK, and PLK1 expression except for CDC2 expression in HCC cells [Figure 4E]. Because cell cycle dysregulation is a distinctive feature of all tumor types and a driving force of tumorigenesis, targeting cell cycle-related proteins is an attractive strategy for effectively halting tumor growth. We therefore tested the anticancer effects of HO-1197 against various human cancer cell lines in an in vitro cell-based assay. The effects of HO-1197 treatment on the viability of lung cancer cells (H460 and H1299), pancreatic cancer cells (Mia-Paca2 and PANC-1), colorectal cancer cells (HT29 and HCT116), and gastric cancer cells (AGS) were similar to its effects on the viability of Huh7 cells [Figure 4F].

### 2.5. HO-1197 Inhibits Angiogenesis and Metastasis in HCC Cells

**Figure 5:**
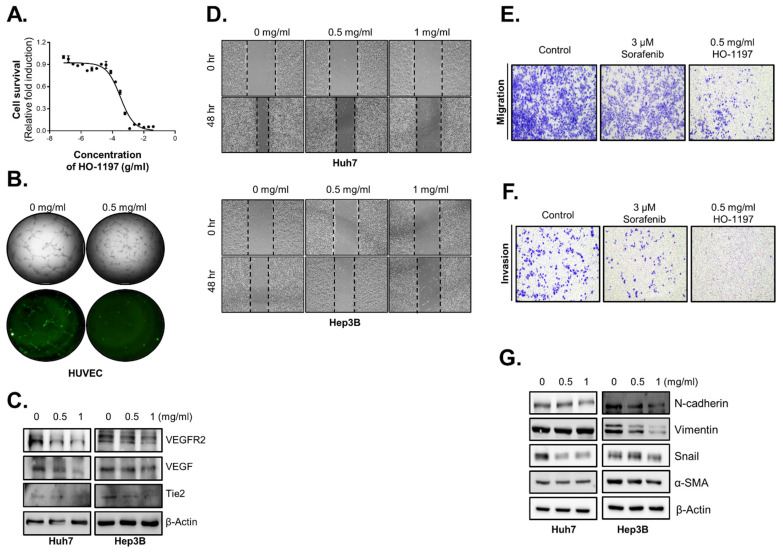
HO-1197 inhibits HCC propagation by attenuating angiogenic and metastatic capacity. (**A**) HUVECs were treated for 48 h with 2-fold sequential serial dilutions of HO-1197 (beginning at 40 mg/mL). (**B**) Capillary tube formation assays of HUVECs treated with 0 or 0.5 mg/mL HO-1197 for 72 h. (**C**) Expression of the angiogenesis-related proteins VEGF, VEGFR2, and Tie2 was examined in Huh7 and Hep3B cells. (**D**) The wound healing capacities of Huh7 and Hep3B cells were examined after treatment with 0, 0.5, or 1 mg/mL HO-1197 for 48 h. (**E**,**F**) Migration (**E**) and invasion (**F**) activity was measured in Huh7 cells treated with 3 μM sorafenib or 0.5 mg/mL HO-1197 using a Transwell system. Cells were stained with crystal violet to visualize migrated or invaded cells. (**G**) The expression of the EMT-related proteins, N-cadherin, Vimentin, Snail, and alpha-smooth muscle actin (α-SMA), was measured in Huh7 and Hep3B cells treated with 0, 0.5, or 1 mg/mL HO-1197 for 48 h. All images were obtained using the HCS system, and values were calculated with Harmony software. Data are expressed as the mean ± SD (n = 3).

Because FoxM1 has also been demonstrated to play essential roles in tumor angiogenesis, invasion, and metastasis [21,22,23], we evaluated changes in the antiangiogenic capacity by HO-1197 treatment using HUVECs. HO-1197 inhibited the proliferation of HUVECs in a dose-dependent manner (IC50 0.32 mg/mL) [Figure 5A]. Next, we evaluated the effects of HO-1197 in an in vitro HUVEC capillary tube formation assay. Compared with PBS-treated cells, HO-1197 treatment reduced HUVEC capillary tube formation on matrigel [Figure 5B]. As FOXM1 directly binds to specific sites on the VEGF gene promoter, enhancing VEGF transcription, we hypothesized that HO-1197, by downregulating FOXM1 activity, may suppress angiogenesis factors. Therefore, we measured the expression of angiogenesis factors, including VEGF, after HO-1197 treatment. Indeed, expression of angiogenesis factors (VEGF, VEGFR3, and Tie-2) was reduced in HO-1197-treated Huh7 and Hep3B cells [Figure 5C].

Because inhibition of angiogenesis is considered a powerful way to suppress tumor metastasis and invasion, we estimated the effects of HO-1197 on the metastatic capacity of HCCs. First, we evaluated HCC migration capacity by introducing a scratch test to assess healing area/wounded area. We found that HO-1197 suppressed the metastatic capacity of Huh7 and Hep3B cells in a dose-dependent manner [Figure 5D]. Additionally, we used transwell migration assays and crystal violet staining to calculate the number of migrated cells after HO-1197 treatment. Treatment with 0.5 mg/mL HO-1197 decreased the number of migrating HCC cells compared with that after treatment with 3 μM sorafenib [Figure 5E]. A transwell invasion assay was performed, and treatment with 0.5 mg/mL HO-1197 inhibited HCC cell invasion [Figure 5F]. As the phenomenon of epithelial–mesenchymal transition (EMT) commonly occurs during tumor metastasis, we measured the expression pattern of classic EMT markers. Western blot analysis revealed that mesenchymal markers (N-cadherin, Vimentin, Snail, and α-SMA) were reduced in HO-1197-treated Huh7 and Hep3B cells [Figure 5G].

### 2.6. HO-1197 Modulates Macrophage Polarization and Enhances NK Cell Activity in the TME

Because numerous studies have demonstrated that traditional herbal medicines enhance immune function in cancer patients through activation of immune cells (e.g., macrophage polarization and NK cell activity) and modulation of the immunosuppressive TME, we also investigated the role of HO-1197 within the immune system of the TME. Considering the possibility that trace amounts of endotoxin potentially present in HO-1197 could affect immune responses, we measured endotoxin levels of HO-1197 prior to conducting functional studies. For 0.0625 mg/mL HO-1197, the endotoxin level was 0.098 EU/mL, and at a higher concentration of 3.125 mg/mL, the level was 0.08 EU/mL. Both values are well below the Ministry of Food and Drug Safety of Korea standard for sterile pharmaceuticals (≤2.5 EU/mL), indicating that HO-1197 contains only minimal endotoxins and is therefore suitable for immune-related experiments.

**Figure 6:**
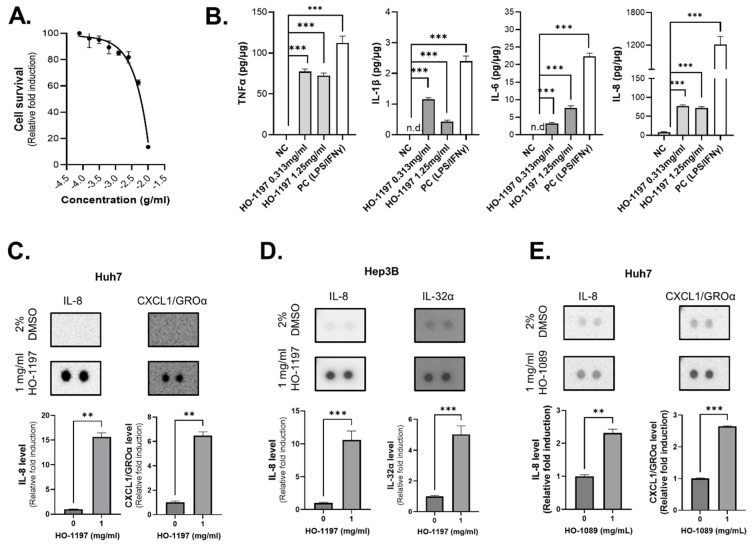
HO-1197 promotes pro-inflammatory cytokine production in the TME. (**A**) THP-1 cells were treated with HO-1197 (starting at 10 mg/mL) using 2-fold serial dilutions for 48 h. Cell survival was calculated by counting nuclei. (**B**) The levels of pro-inflammatory cytokines (TNF-α, IL-1β, IL-6, and IL-8) secreted from THP-1 cells treated with 0, 0.313, or 1.25 mg/mL HO-1197 were detected using ELISAs. (**C**–**E**) A cytokine array was performed (upper), and cytokine levels were quantified (lower) in Huh7 (**C**) and Hep3B (**D**) cells treated with HO-1197 (0 or 1 mg/mL) and in Huh7 cells treated with HO-1089 (0 or 1 mg/mL) (**E**). Data are expressed as the mean ± SD (n = 3). ** *p* < 0.005 and *** *p* < 0.0005 compared with the control group.

HO-1197 concentrations exceeding 2.5 mg/mL significantly promoted THP-1 cell death [Figure 6A]. THP-1 cells were incubated with lipopolysaccharide and IFN-γ as a positive control for M1 polarization, leading to the production of pro-inflammatory cytokines. HO-1197 treatment at concentrations less than 2.5 mg/mL promoted the secretion of pro-inflammatory cytokines, such as TNF-α, IL-1β, IL-6, and IL-8, in THP-1 cells. [Figure 6B]. To investigate the influence of HO-1197 on the TME, we also screened human cytokine expression in HO-1197-treated Huh7 and Hep3B cells. HO-1197 commonly increased IL-8 expression in both Huh7 and Hep3B cells while specifically upregulating CXCL1 in Huh7 cells and IL-32α in Hep3B cells [Figure 6C,D]. Moreover, HO-1197 treatment more effectively upregulated IL-8 expression in Huh7 cells than HO-1089 [Figure 6E].

**Figure 7:**
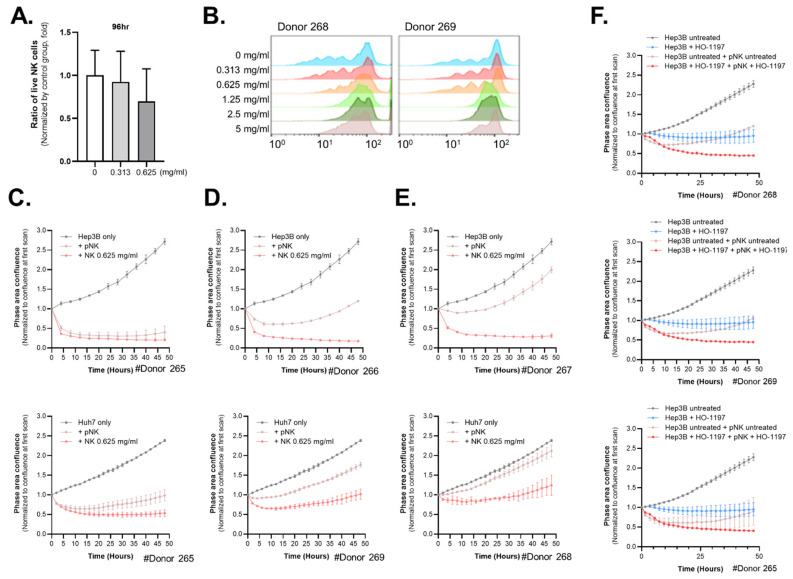
HO-1197 enhances NK cell antitumor activity. (**A**) The percentages of live NK cells after treatment with HO-1197 (0, 0.313, or 0.625 mg/mL) were measured. (**B**) Measurement of CellTrace Violet (CTV) dilutions in NK cells treated with 0, 0.313, 0.625, 1.25, 2.5, or 5 mg/mL HO-1197 for donors 268 and 269. (**C**–**E**) Activated NK cells were pre-incubated with 0.0625 mg/mL HO-1197 and co-cultured with Hep3B (upper) or Huh7 (lower) cells. NK cells were classified into three groups according to their intrinsic cytotoxic responses against Hep3B and Huh7 cells: high (**C**), intermediate (**D**), and low (**E**). (**F**) Hep3B and NK cells were co-treated with 0.625 mg/mL HO-1197. The phase area confluence of Hep3B cells was measured to evaluate their susceptibility to the cytotoxic activity of NK cells.

Next, we explored the effects of HO-1197 on NK cells, which are immune cells with natural antitumor activity. First, NK cells derived from four different healthy donors were treated with HO-1197 concentrations ranging from 0.3125 to 10 mg/mL to assess its impact on NK cell survival and proliferation. At a concentration of 0.625 mg/mL, cell viability was markedly reduced after 4 days, although the remaining cells retained proliferative capacity. Concentrations ≥ 1.25 mg/mL induced pronounced cytotoxicity, characterized by severe morphological alterations and cessation of proliferation [Figure 7A,B]. To assess the effects of HO-1197 on their cytotoxic capacity, NK cells were incubated with 0.0625 mg/mL HO-1197, washed, and co-cultured with two target cell lines (Hep3B and Huh7). The anti-tumor activity of NK cells exhibited donor-dependent variability. To investigate the effect of HO-1197 on NK cell with divergent killing capacity, we classified NK cells based on their intrinsic antitumor activity against Hep3B and Huh7 cells into three groups: high [Figure 7C], intermediate [Figure 7D], and low [Figure 7E]. Following HO-1197 treatment, NK cells with high baseline activity showed a minimal increase in cytotoxic function, while a significant enhancement was observed in the intermediate group, and the most pronounced increase was seen in the low-activity group [Figure 7C–E]. These results suggest that HO-1197 can improve NK cell antitumor, with the HO-1197 particularly enhancing the function of NK cells with low intrinsic antitumor activity. NK cells pretreated with HO-1197 demonstrated potent cytotoxic activity against HCC cell lines, including both Hep3B and Huh7 cells, although the degree of antitumor efficacy varied between these cell lines. Hep3B cells were nearly completely eradicated following 48 h of co-culture with NK cells pretreated with HO-1197, whereas a considerable proportion of Huh7 cells remained viable under the same conditions.

Next, we observed the effect of concurrent exposure of HCCs and NK cells to 0.625 mg/mL HO-1197 on HCC susceptibility to NK cell-mediated cytotoxicity. While HO-1197 treatment or co-culture with NK cells alone led to substantial elimination of Hep3B cells, a pronounced synergistic antitumor effect was observed upon simultaneous incubation of NK cells and Hep3B cells in the presence of HO-1197 [Figure 7F]. However, when the same experiment was performed using Huh7 cells instead of Hep3B cells, HO-1197 treatment or co-culture with NK cells resulted in lower antitumor efficacy compared to Hep3B cells. Moreover, no significant synergistic antitumor effect was observed upon simultaneous incubation of NK cells with Huh7 cells in the presence of HO-1197 [Supplementary Figure S8]. These results suggest that slight variations in immune responses to HO-1197 may occur among patients with HCC.

### 2.7. Combination of HO-1197 and Sorafenib Produces Synergistic Antitumor Effects in HCCs

**Figure 8:**
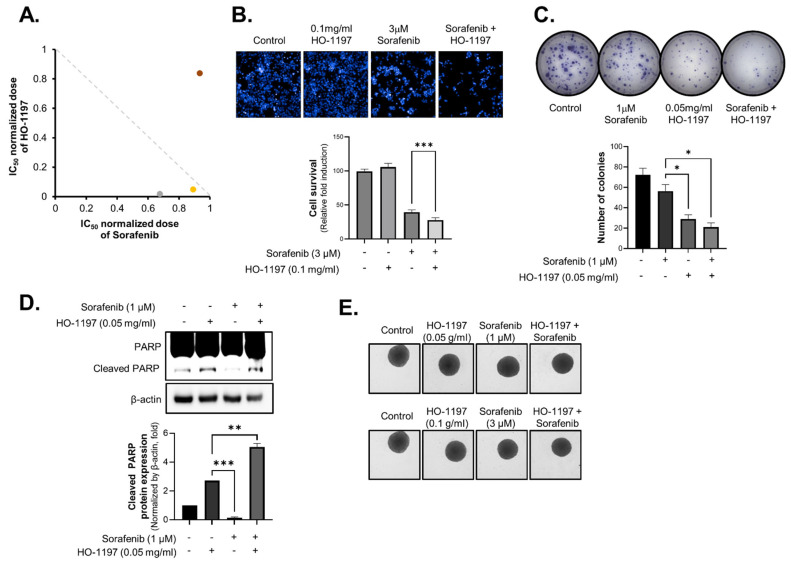
The combination of HO-1197 and sorafenib enhances antitumor effects. (**A**) Combination index (CI) values were calculated in Huh7 cells co-treated with sorafenib and HO-1197. (**B**) Hep3B cells were treated with 3 μM sorafenib, 0.1 mg/mL HO-1197, or co-treated with sorafenib (3 μM) and HO-1197 (0.1 mg/mL). Cell survival was analyzed by nucleus staining (upper) and calculated (lower). Images were obtained using the HCS system, and values were calculated with Harmony software. (**C**,**D**) Huh7 cells were treated with 3 μM sorafenib, 0.1 mg/mL HO-1197, or co-treated with sorafenib (1 μM) and HO-1197 (0.1 mg/mL). A colony formation assay was performed by staining with crystal violet (**C**), and apoptosis marker (cleaved PARP) expression was measured by immunoblotting (**D**). (**E**) Immune-activated multicellular HCC spheroids were co-treated with sorafenib and HO-1197. Spheroid size and morphology were analyzed using Harmony software. Data are expressed as the mean ± SD (n = 3). * *p* < 0.05, ** *p* < 0.005, *** *p* < 0.0005 compared with the control group.

The combination of anticancer agents and traditional herbal medicines can synergistically enhance tumor suppression, improve immune function, reduce side effects, and potentially overcome drug resistance, providing a more comprehensive and patient-centered cancer treatment strategy. We evaluated the combinatory effects of the angiogenesis inhibitor sorafenib and HO-1197 using combination index (CI) analysis. Notably, co-treatment with sorafenib and HO-1197 synergistically inhibited Huh7 cell proliferation. The CI values ranged from 0.2 to 0.7, indicating a synergistic interaction. A dose-normalized isobologram was constructed to assess the interaction between the two drugs. The strongest synergistic effect was observed with 1 μM sorafenib and 0.05 mg/mL HO-1197, with a CI50 of 0.695 [Figure 8A].

Compared with sorafenib treatment alone, combination treatment with sorafenib and HO-1197 exhibited markedly superior efficacy, demonstrating significantly greater inhibition of HCC cell growth [Figure 8B], more pronounced suppression of colony formation [Figure 8C], and substantially greater induction of apoptosis in HCC cells [Figure 8D].

To further investigate the synergistic effects of this combination therapy, we utilized an immune-activated multicellular HCC spheroid model comprising HCC cells, hepatic stellate cells, vascular endothelial cells, fibroblasts, and macrophages. Co-treatment with sorafenib and HO-1197 led to a substantial reduction in spheroid size and exhibited enhanced antitumor activity compared with sorafenib treatment alone [Figure 8E].

## 3. Discussion

HCC typically arises from chronic liver inflammation, fibrosis, and cirrhosis, evolving into a biologically complex and clinically challenging malignancy characterized by intratumoral heterogeneity, immune evasion mechanisms, and resistance to conventional therapeutic interventions. Although the Food and Drug Administration has approved two major classes of drugs for HCC treatment—angiogenesis inhibitors and immune checkpoint inhibitors—these monotherapies remain insufficient to fully address the complex and aggressive nature of the disease. Recently, combination therapies involving immune checkpoint blockade and antiangiogenic agents have shown improved clinical responses compared with those of conventional treatments, offering a promising direction for systemic HCC therapy. However, the optimal drug combinations and mechanisms of synergy remain poorly defined.

In this context, herbal medicine has a long history of use for the treatment of numerous diseases. Various herbal formulas have been used for many years to treat HCC with good efficacy and safety, mainly in East Asia, despite a lack of high-quality, multicenter clinical trials [12,24]. Many traditional herbal formulas exert multi-targeted effects, including anti-inflammatory, antifibrotic, immunomodulatory, and anticancer activities, which align well with the multifaceted pathology of HCC [24]. Investigating these natural compounds not only broadens the therapeutic landscape but also provides opportunities to identify safe and effective agents that can be integrated into combination regimens [25]. Our study aims to explore this potential by evaluating the monotherapeutic efficacy and mechanisms of a novel herbal formulation in HCC models, as well as its combinatorial efficacy.

To this end, we investigated the anticancer properties of HO-1197, a reconstituted herbal formulation derived from HO-1089, and revealed its multifaceted mechanisms of action across cellular, molecular, and immunological axes. By integrating transcriptomic profiling, functional assays, and immune-activated three-dimensional spheroid models, we provide compelling evidence that HO-1197 exerts its therapeutic effects through the induction of ROS-mediated DNA damage, FoxM1-targeted transcriptional disruption, and remodeling of the tumor immune microenvironment.

Mechanistically, HO-1197 induced robust DNA damage Via ROS generation, leading to G2/M phase arrest irrespective of p53 status. This mechanism is particularly significant given the prevalence of TP53 mutations in approximately one-third of HCC cases. HO-1197 circumvented p53 dependency by activating p21Cip1/Waf1 independently, offering a therapeutic avenue for tumors that are typically resistant to DNA damage-induced checkpoint arrest. In contrast to conventional ROS-inducing agents, HO-1197 demonstrated enhanced selectivity for cancer cells while sparing non-malignant hepatocytes, supporting its favorable therapeutic index. Notably, ROS inducers such as NCX4040 (a nitric oxide donor) have shown similar efficacy by depleting glutathione and amplifying oxidative DNA damage [26], while platinum(II) metallacycles selectively target folate receptor-overexpressing cancer cells Via ROS-dependent mechanisms [27].

One of the most striking findings of this study is the ability of HO-1197 to downregulate FOXM1—a transcription factor recognized as a critical driver of HCC proliferation, stemness, and therapy resistance. Transcriptomic analysis revealed widespread downregulation of FOXM1 target genes involved in mitotic progression, such as PLK1, AURKA, and CENPA. This FOXM1 suppression not only impaired nuclear translocation but also destabilized mitotic checkpoint fidelity [28,29], resulting in SAC failure [30] and mitotic catastrophe. These events collectively disrupt the proliferative machinery of HCC cells and highlight FOXM1 as a pivotal molecular vulnerability targeted by HO-1197. Although our study demonstrated that HO-1197 suppresses FOXM1 mRNA expression and inhibits its nuclear localization, we did not perform direct promoter or transcriptional assays. Future studies will investigate whether HO-1197 or its single active components directly modulate FOXM1 transcriptional activity. Similar FOXM1 inhibition strategies have been reported for Artemisinin, which disrupts the interaction of FOXM1 with promoter regions of oncogenic drivers such as PLK1 and AURKA [31], and for STL001, a small-molecule FOXM1 inhibitor that synergizes with PARP inhibitors in homologous recombination-deficient tumors [32].

Beyond its direct antitumor effects, HO-1197 displayed notable antiangiogenic and antimetastatic activity by modulating the FOXM1–VEGF/VEGFR2–Snail axis through regulation of FOXM1 expression. Because the FOXM1–VEGF/VEGFR2–Snail axis coordinates tumor growth, angiogenesis, and metastasis, targeting this pathway is a promising anticancer strategy [33,34].

HO-1197 inhibited key mesenchymal markers, impaired endothelial tube formation, and attenuated VEGF/VEGFR2 and Snail expression—suggesting that it may disrupt tumor-stroma crosstalk and vascular support systems essential for metastatic progression. These properties are particularly relevant in the context of advanced HCC, where angiogenesis is a hallmark feature of aggressive disease. Recent studies highlight that FOXM1 inhibitors, when combined with anti-VEGFR2 agents, enhance angiogenesis suppression in HCC xenografts [32], mirroring the dual targeting observed for HO-1197.

The immunomodulatory effects of HO-1197 further underscore its potential as a systemic agent. By enhancing NK cell-mediated cytotoxicity—especially in donor-derived NK cells with low baseline activity—HO-1197 addresses the clinical challenge of postoperative recurrence, which remains a major cause of HCC-related mortality [35,36]. Notably, the degree of NK cell activation following HO-1197 treatment varied among donors, likely due to differences in baseline activation state and genetic background. This suggests that HO-1197 is particularly beneficial for individuals with intrinsically low NK cell cytotoxicity. Its capacity to prime innate immune responses through M1 macrophage polarization and cytokine upregulation (e.g., TNF-α, IL-1β, IL-6, IL-8) suggests that HO-1197 can reprogram the immunosuppressive HCC microenvironment toward a pro-inflammatory and immunostimulatory state [37]. This capacity aligns with clinical strategies combining CSF1R inhibitors (to reduce M2 macrophages) with PD-1/CTLA-4 blockade, which enhance cytotoxic T-cell infiltration [38].

HO-1197 demonstrated potent antitumor efficacy as a monotherapy. In parallel, HO-1197 synergized with sorafenib, the long-standing standard-of-care tyrosine kinase inhibitor. This combination reduced spheroid volumes significantly more than did sorafenib treatment alone, with CI50 values indicating robust synergy. Given that sorafenib has been reported to paradoxically induce FOXM1 upregulation—a mechanism associated with treatment resistance—the ability of HO-1197 to suppress FOXM1 may provide a rational basis for this observed synergy. The synergy of HO-1197 with sorafenib mirrors broader trends in herbal anticancer drug research. Herbal formulations such as Shi-Quan-Da-Bu-Tang, which enhances hematopoietic activity and reduces chemotherapy-induced side effects through immunomodulation, have demonstrated synergy with cisplatin in preclinical models, reducing tumor growth by over 40% [39]. Chebulae Fructus induces ROS-mediated apoptosis while modulating the TGF-β/MAPK pathway, with additive effects when combined with 5-fluorouracil [40]. Similarly, Salvia miltiorrhiza enhances doxorubicin efficacy by downregulating Bcl-2, activating caspases-3/9, and inhibiting drug efflux pumps such as P-glycoprotein [41].

Despite these promising findings, several limitations warrant consideration. The bioactive constituents of HO-1197, such as curcuminoids and bornyl acetate, require further fractionation and pharmacokinetic validation to determine the components most critical for efficacy. NK cell responses to HO-1197 varied across donors, highlighting the need for immune biomarkers to guide patient stratification and maximize clinical benefit.

## 4. Materials and Methods

### 4.1. Preparation of HO-1197

To investigate the combined effects of traditional herbal ingredients, HO-1197 was formulated using *H. herba*, *A. fructus*, *C. zedoaria* Rosc., Curcuma root, and prunella spike. All herbs were acquired from Yeonhcheon Herbal Market (Yeongcheon, Republic of Korea), and their authenticity and quality were verified by H&O Biosis (Hwaseong-si, Republic of Korea). For extraction, precisely 150 g of the herbal blend were immersed in 1 L of distilled water and brought to a vigorous boil for 2 h using a 2 L glass flask (Saehanlab Co., Seoul, Republic of Korea). The resulting decoction was cooled to room temperature and sequentially filtered: initially through an 8-µm polyester filter (Hwa Shin Textile Filter Co., Eumseong-si, Republic of Korea), followed by further clarification with a 0.5-µm polypropylene cartridge (Teail Industries Co., Hwaseong-si, Republic of Korea). The filtrate was transferred to a freeze dryer, lyophilized, and the powder stored in a vacuum desiccator at 4 °C to ensure sample stability. For experimental use, the powder was dissolved in pre-heated distilled water, and the concentration was adjusted according to specific assay requirements.

### 4.2. Cell Lines and Culture Conditions

This study utilized a broad panel of human cancer cell lines: hepatocellular carcinoma lines (Huh7, Hep3B, SNU449, SNU475, PLC/PRF/5, HepG2, Huh6), lung cancer (H460, H1299), pancreatic cancer (Mia-Paca-2, PANC-1), colon cancer (HT29, HCT116), and gastric cancer (AGS), enabling comprehensive evaluation of HO-1197’s anti-tumor activity. Additionally, hepatic stellate cells (LX2), human lung fibroblasts (WI38), monocytic leukemia line (THP-1), and immortalized human hepatocytes (Fa2N-4) were included to assess assay specificity and off-target effects. Cell authentication was confirmed by the Korean Cell Line Bank, with provenance and passage numbers tracked. Huh6 was generously provided by Dr. Ralf Bartenschlager (University of Heidelberg). LX2 was purchased from Merck Millipore; WI38 and THP-1 from ATCC; and Fa2N-4 from Xenotech (Lenexa, KS, USA). Cells were seeded according to experimental design and cultured in their recommended media: RPMI 1640 (Welgene, Gyeongsan-si, Republic of Korea) for Huh7, SNU449, SNU475, PLC/PRF/5, H460, H1299, AGS, and THP-1 (with 25 mM HEPES and 50 μM 2-mercaptoethanol); MEM (Welgene, Republic of Korea) for Hep3B and WI38; and DMEM (Welgene) for HepG2, Huh6, Mia-Paca-2, PANC-1, HT29, and HCT116. Fa2N-4 cells were plated in serum-containing medium and transitioned to supporting medium post-attachment (Xenotech protocol).

All media were supplemented with 10% heat-inactivated fetal bovine serum (Gibco), 1× penicillin/streptomycin (Gibco), and maintained in a humidified incubator at 37 °C, 5% CO_2_. Passage number was kept below 30 for all experiments to minimize genetic drift.

For 3D spheroid formation, HCC, LX2, WI38, HUVEC, and THP-1 cells were seeded at 6 × 10^3^/well into 96-well, ultra-low attachment round-bottom microplates (Corning Life Sciences, Corning, NY, USA) and allowed to aggregate prior to treatment.

### 4.3. Dose–Response Curves

To analyze cellular sensitivities to HO-1197 and sorafenib, HCC, normal hepatocytes (Fa2N-4), hepatic stellate cells (LX2), human lung fibroblasts (WI38), and additional cancer lines were seeded at 2 × 10^3^ cells/well in 384-well plates (Greiner Bio-One, Monroe, NC, USA). After adherence, cells were exposed to HO-1197 (up to 40 mg/mL, serial 2-fold dilution across 10 concentrations in water) or sorafenib (up to 20 μM, serial 2-fold dilution in DMSO), maintaining constant vehicle proportions. Incubation was performed for 48 h. Following treatment, cells were fixed in 4% paraformaldehyde (Sigma-Aldrich, St. Louis, MO, USA) at room temperature for 10 min, washed with DPBS (Welgene, Gyeongsan-si, Republic of Korea), and nuclei stained with Hoechst 33,342 (20 μg/mL, Invitrogen, Waltham, MA, USA) for 10 min. Automated image acquisition was performed using the Operetta High-Content Screening system (PerkinElmer, Shelton, CT, USA), and cell counts were normalized to untreated controls across five random fields per well. Dose–response curves were fitted with non-linear regression models to derive IC_50_ values.

### 4.4. Colony Formation Assay

Anchorage-dependent growth was assessed by seeding Huh7 and Hep3B cells at low density (1 × 10^3^/well) in 6-well plates, followed by exposure to HO-1197 (0.5 and 1 mg/mL) for a 14-day period. Media was replenished bi-weekly. Colonies formed were fixed in 4% paraformaldehyde (Biosesang, Yongin-si, Republic of Korea), stained with 0.5% crystal violet, and thoroughly washed. Colonies exceeding 50 cells were counted using the AID vSpot Spectrum system (Autoimmun Diagnostika GmbH, Strassberg, Germany).

### 4.5. Protein Separation and Immunoblot Analysis

For protein profiling, cells were lysed in ice-cold RIPA or proprietary lysis buffer (Thermo Fisher Scientific, Waltham, MA, USA) for 30 min, sheared, and clarified by centrifugation (13,200 rpm, 4 °C, 10 min). Supernatants were mixed with 5× sample buffer (BioSolutions, Seoul, Republic of Korea), denatured at 95 °C for 10 min, and equal aliquots (10–30 μg) loaded onto 8% or 10% SDS-polyacrylamide gels. After electrophoresis, proteins were transferred to nitrocellulose membranes (Pall Corp., Port Washington, WI, USA), blocked with 5% skim milk (BD Biosciences, Franklin Lakes, NJ, USA), and incubated overnight at 4 °C with primary antibodies targeted to CD133/1, PARP, cleaved caspase-3, total caspase-3, γ-H2AX, p21, p-p53(S15), FoxM1, Aurora A, Bub1, TTK/Mps1, PLK1, CDC2, VEGF, VEGFR2, Tie2, N-cadherin, Vimentin, Snail, α-SMA, and β-actin (details in Supplementary Table S1). Following DPBS washes, blots were incubated with HRP-conjugated secondary antibodies and developed using enhanced chemiluminescence (ECL) reagents. Images were acquired on X-Omat AR film (Eastman Kodak, Rochester, NY, USA). β-actin served as loading control for normalization.

### 4.6. Cell Cycle Analysis

Cellular DNA content distribution was assessed by treating Huh7 cells with HO-1197 (0, 1 mg/mL) for 48 h. Cells were harvested, washed in DPBS, fixed in 70% ethanol (Sigma-Aldrich, St. Louis, MO, USA) on ice, and incubated with RNase A at a final concentration of 100 μg/mL (using a stock solution prepared in PBS) for 30 min. After that, the lysates stained with 5 μL propidium iodide (Sigma-Aldrich) in DPBS for 10 min. Flow cytometric analysis (BD Biosciences) was performed to determine the percentage of cells in subG1, G1, S, and G2/M phases.

### 4.7. Reactive Oxygen Species Detection

Intracellular ROS generation was measured in Huh7, Hep3B, LX2 and WI38 cells (2 × 10^3^/well, 384-well plate) following HO-1197 (0, 0.5 mg/mL) exposure for 24 h. Cells were washed, then incubated with CM-H2DCFDA (Thermo Fisher Scientific, Waltham, MA, USA) for 30 min at 37 °C with Hoeschst 33,342 for nuclei staining. Fluorescence imaging was performed with the Operetta system, using specific excitation (485 ± 20 nm) and emission (515 ± 10 nm) settings. Alexa 488 intensity and Hoechst positive cell numbers were quantified, and ROS intensity was normalized to cell number.

### 4.8. Immunocytochemistry

For subcellular localization studies, Huh7 or Hep3B cells (2 × 10^3^/well) were seeded, incubated with HO-1197 (0, 0.5, 1 mg/mL) for 48 h, and fixed at room temperature. Cells were permeabilized and blocked in 10% goat serum and 0.1% Triton X-100, then incubated with rabbit anti-FoxM1 antibody (Abcam, Cambridge, UK) overnight at 4 °C. After DPBS washes, secondary fluorescent antibody (Invitrogen) incubation proceeded for 2 h. Nuclei were counterstained with Hoechst 33,342. High-content imaging and automated quantification were performed to determine nuclear FoxM1 expression.

### 4.9. Next-Generation Sequencing

Total RNA was isolated from Huh7 cells treated with 1 mg/mL HO-1197, and its concentration was determined using the Quant-IT RiboGreen assay (Invitrogen). RNA integrity was evaluated with the RNA ScreenTape system on a TapeStation instrument (Agilent Technologies, Santa Clara, CA, USA), and only samples with an RNA integrity number (RIN) above 7.0 were selected for further library preparation. For each sample, 1 µg of high-quality RNA was subjected to library construction using the Illumina TruSeq Stranded mRNA Sample Prep Kit (Illumina, San Diego, CA, USA). Poly-A–containing transcripts were first captured with poly-T–conjugated magnetic beads. The enriched mRNA was fragmented into short fragments under high-temperature conditions with divalent cations. First-strand cDNA was synthesized using random hexamer primers and SuperScript II reverse transcriptase (Invitrogen), followed by second-strand cDNA synthesis with DNA polymerase I, RNase H, and dUTP incorporation. The resulting cDNA fragments underwent end repair, the addition of a single ‘A’ base at the 3′ ends, and adaptor ligation. PCR amplification and purification steps were then carried out to generate the final cDNA libraries. Library concentration was assessed with the KAPA Library Quantification Kit for Illumina platforms using the qPCR-based method (KAPA BIOSYSTEMS, Wilmington, MA, USA), and fragment size distribution was verified with the TapeStation D1000 ScreenTape (Agilent Technologies). Indexed libraries were subsequently subjected to paired-end sequencing (2 × 100 bp) on the Illumina NovaSeq X platform (Illumina) at Macrogen Incorporated.

### 4.10. Quantitative Real-Time PCR

Huh7 and Hep3B cells were treated with HO-1197 (0, 0.5 mg/mL), and RT–PCR performed using an iScript cDNA Synthesis Kit (Bio-Rad, Hercules, CA, USA). Primer sequences are shown in Supplementary Table S1. qPCR was run with SYBR Green in a StepOnePlus system (Applied Biosystems, Waltham, MA, USA); CT values normalized to β-actin using the 2^−ΔΔCT^ model. Each reaction was performed in triplicate.

### 4.11. Data Processing and Analysis

Sequencing data were obtained as paired-end reads from the Illumina NovaSeq X platform. Prior to downstream analysis, adapter sequences and low-quality bases were filtered out using Trimmomatic v0.38. The cleaned reads were mapped to the *Homo sapiens* GRCh38 genome using HISAT v2.1.0, which incorporates the HISAT and Bowtie2 algorithms. The genome assembly and annotation files were retrieved from the NCBI Genome Assembly and NCBI RefSeq databases. Alignment outputs in SAM format were subsequently sorted and indexed with SAMtools v1.9. Transcript assembly and quantification were then carried out with StringTie v2.1.3b. Gene- and transcript-level expression values were reported as raw read counts, FPKM (fragments per kilobase of transcript per million mapped reads), and TPM (transcripts per million).

### 4.12. Differential Gene Expression Analysis

Differential gene expression analysis was conducted using edgeR v3.40.2 with raw read counts as the input. During quality control, only genes expressed with non-zero counts across all samples were retained. To evaluate the similarity of expression patterns among samples, principal component analysis (PCA) and multidimensional scaling (MDS) were applied. Normalization of the dataset was carried out using the trimmed mean of M-values (TMM) method to adjust for differences in library sizes. Differentially expressed genes were identified through the exactTest function in edgeR, and the resulting fold-change values and *p*-values were obtained. All *p*-values were adjusted using the Benjamini–Hochberg approach to estimate the false discovery rate (FDR). Genes were considered significantly altered when |fold change| ≥ 2 and raw *p*-value < 0.05. For significant genes, hierarchical clustering of rlog-transformed values was conducted with the parameters: distance metric = Euclidean distance and linkage method = complete. Gene enrichment and functional annotation analysis of significant genes was performed using gProfiler (https://biit.cs.ut.ee/gprofiler/orth (accessed on 17 October 2023)) with reference to the Gene Ontology database. gProfiler enrichment *p*-values were calculated using a one-sided hypergeometric test and further adjusted by the Benjamini–Hochberg method. All statistical analyses and visualization of differential gene expression results were performed in R version 4.2.2 (www.r-project.org (accessed on 17 October 2023)).

### 4.13. Wound Healing Assays

Huh7 or Hep3B cells were plated in 6-well plates (Corning) at a density of 1 × 10^6^ cells per well and cultured until they formed a confluent monolayer. Once confluence was achieved, a scratch was introduced across the monolayer using a yellow pipette tip. Cells were then treated with HO-1197 at concentrations of 0, 0.5, or 1 mg/mL. The closure of the wound area was monitored for 24 h, and images were acquired using a light microscope (Zeiss, Jena, Germany).

### 4.14. Transwell Migration Assays

Cell migration was assessed using 6.5 mm Transwell^®^ chambers containing 8.0 µm pore polycarbonate membranes (Corning). In the upper chamber, 100 µL of Huh7 cells suspended in DMEM supplemented with 2% fetal bovine serum (FBS) were seeded at a density of 5 × 10^3^ cells/well. The lower chamber was filled with 500 µL of DMEM containing 10% FBS. Sorafenib (3 µM) and HO-1197 (0.5 mg/mL) were applied during the 48 h incubation. Following incubation, migrated cells on the underside of the membrane (lower surface of the upper chamber) were fixed with 4% paraformaldehyde for 10 min and stained with 0.05% crystal violet. Non-migrated cells on the upper surface of the membrane were carefully removed with a cotton swab. Images of stained cells were captured by microscopy and analyzed using OptiView software (version 4.12, Republic of Korea Lab Tech, Seoul, Republic of Korea).

### 4.15. Transwell Invasion Assays

The invasion assay was performed as described in Section 4.14. For the invasion assay, the upper chamber of the Transwell was coated with 100 µL of Matrigel (Corning) diluted in serum-free DMEM and incubated for 24 h at 37 °C in a 5% CO_2_ incubator before cell seeding.

### 4.16. Cytotoxicity in THP-1 Macrophages

THP-1 cells (DSMZ, Braunschweig, Germany) were seeded into 96-well plates at a density of 5 × 104 cells/100 μL with 100 ng/mL PMA (Sigma-Aldrich, St. Louis, MO, USA) for macrophage differentiation. The cells were incubated for 24 h, followed by 24 h of incubation with HO-1197 at the following concentrations: 10, 5, 2.5, 1.25, 0.625, 0.313, and 0.156 mg/mL. Cell metabolic activity was measured using an XTT assay (Sigma–Aldrich), and the results are expressed as mitochondrial dehydrogenase activity as a percentage (%) of the negative control. Additionally, the total protein amount per well (μg/well) was measured using a BCA assay (Thermo Fisher Scientific). The assay was accepted according to assay acceptance criteria.

### 4.17. Inflammatory Effects on THP-1 Macrophages

THP-1 cells (DSMZ, Braunschweig, Germany) were seeded into 96-well plates at 1.5 *×* 10^4^ cells/150 µL with 100 ng/mL PMA (Sigma-Aldrich) for 24 h, followed by a 24 h incubation without PMA. After that, cells were incubated with the formula HO-1197 in the concentrations: 10, 5, 2.5, 1.25, 0.625, 0.313 or 0.156 mg/mL for 24 h. A positive control incubated for 24 h with 100 ng/mL LPS (Sigma Aldrich) and 20 ng/mL IFNγ (Thermo Fisher Scientific) for inflammatory stimulation of the macrophages was included in the assay. The assay was accepted according to assay acceptance criteria. After the incubation with the formula HO-1197 cell culture supernatants were harvested for ELISA analysis and cells were lysed using RIPA buffer (150 nM NaCl, 50 mM TRIS, 0.5% sodium deoxycholate, 0.1% SDS, 1% Triton-x 100) supplemented with protein inhibitor cocktail (Sigma Aldrich) for total cell protein determination.

### 4.18. ELISA Analysis

Secreted cytokines were measured using a series of commercially available ELISA kits, including Human TNF-alpha DuoSet ELISA, Human IL-1 beta/IL-1F2 DuoSet ELISA, Human IL-6 DuoSet ELISA, and Human IL-8/CXCL8 DuoSet ELISA (all from R&D Systems, Minneapolis, MN, USA). To normalize cytokine levels, total protein content in each sample was determined using the BCA Protein Assay Kit (Thermo Fisher Scientific). All assays were conducted following the manufacturers’ detailed protocols, and the results were evaluated against the recommended quality and acceptance criteria to ensure the reliability of the measurements.

### 4.19. Cytokine Array

A Human Cytokine Array (R&D Systems) was used to quantify pro-inflammatory cytokines in cell lysates, according to the manufacturer’s protocol. Protein was extracted as described in the protein separation section, and its concentration was determined using a BCA assay. In brief, nitrocellulose membranes were exposed to the Huh7 and Hep3B cell lysates. An antibody detection cocktail and streptavidin-horseradish peroxidase were used to detect cytokines, and chemiluminescent detection reagents were applied to develop the blots. The blots were quantified and analyzed using Chemi Fluoro Imager software (version AF9S, Davinch-K, Seoul, Republic of Korea).

### 4.20. Natural Killer (NK) Cell Isolation and Culture

Peripheral blood samples from healthy volunteers were used for NK cell isolation with the RosetteSep Human NK Cell Enrichment Cocktail (STEMCELL Technologies, Vancouver, BC, Canada). Written informed consent was obtained from all participants, and the procedure was conducted in accordance with the Declaration of Helsinki and approved by the Institutional Research Ethics Committee of the University Clinic Leipzig (approval number 327/22-ek). Isolated NK cells were maintained in NK MACS medium (Miltenyi Biotec) supplemented with 5% human serum (Sigma–Aldrich), IL-2 (500 U/mL), and IL-15 (140 U/mL) (PeproTech, Rocky Hill, NJ, USA). Cultures were incubated at 37 °C in a humidified atmosphere with 5% CO_2_ for 4–18 days.

### 4.21. CellTrace Violet Celll Proliferation Assay

CellTrace™ Violet (Invitrogen) stock solution with a concentration of 5 mM was prepared by adding 20 μL of DMSO to one vial of CellTrace™ reagent. For a working concentration of 1 μM, stock solution was diluted 1:5000 in PBS. NK cells were resuspended in 1 mL of 1 μM CellTrace Violet working solution, ensuring a cell concentration of up to 1 × 10^6^ cells/mL for the staining volume. NK cells were incubated for 20 min at 37 °C and protected from light. Staining was stopped by adding complete culture medium (containing at least 1% protein) to the cells and incubating for an additional 5 min. The cells were pelleted and then resuspended in fresh complete culture medium (NK MACS supplemented with cytokines) with varying amounts of HO-1197 extract. Cells were seeded by adding equal amounts of cells per well in a total volume of 150 μL on a 96-well suspension plate at densities ranging from 6.5 × 10^4^ to 2.5 × 10^5^ cells per donor/well. The cells were incubated at 37 °C and 5% CO_2_ for 4 days until cell proliferation and survival were analyzed.

### 4.22. Assessment of Target Cell Sensicivity

Target cell confluence was measured using the Basic Analyzer software (version v2024B) of the Incucyte Live Cell Analysis Instrument (Sartorius, Göttingen, Germany). Measurements were performed for at least 48 h in the adherent cell-by-cell mode with 10× magnification and four images per well. Data were normalized to the confluence of the first scan.

### 4.23. Fractional Inhibitory Concentration (FIC)

For the combination treatment, cells were incubated with HO-1197 and sorafenib (Santa Cruz, CA, USA) for 48 h. The maximum concentration of HO-1197 was 20 mg/mL (2-fold dilution, 10 points) in water, and sorafenib was 50 μM (2-fold dilution, 10 points) in DMSO. The synergistic effects of the two compounds were evaluated using the Fractional Inhibitory Concentration (FIC) index. The combination index (CI) values were obtained using Compusyn software (version 1.0) to identify synergistic (CI < 1), additive (CI = 1), or antagonistic interactions (CI > 1).

### 4.24. Endotoxin Detection

The Pierce Chromogenic Endotoxin Quant Kit (Thermo Fisher Scientific) was used to quantify endotoxin in HO-1197 compound, following the manufacturer’s instructions. Compounds and endotoxin standards were incubated with reconstituted Amebocyte Lysate Reagent supplied in the kit at 37 °C. A chromogenic substrate was applied to detect endotoxin, followed by the addition of a stop solution (25% acetic acid). Endotoxin levels in the compounds were calculated based on a standard curve.

### 4.25. Statistical Analysis

All experiments were performed at least three times, and the data are presented as the mean ± SD. We used Student’s *t*-test in Microsoft Excel to determine statistical significance, which is reported using the following notations: * *p* < 0.05, ** *p* < 0.005, and *** *p* < 0.001.

## 5. Conclusions

**Figure 9:**
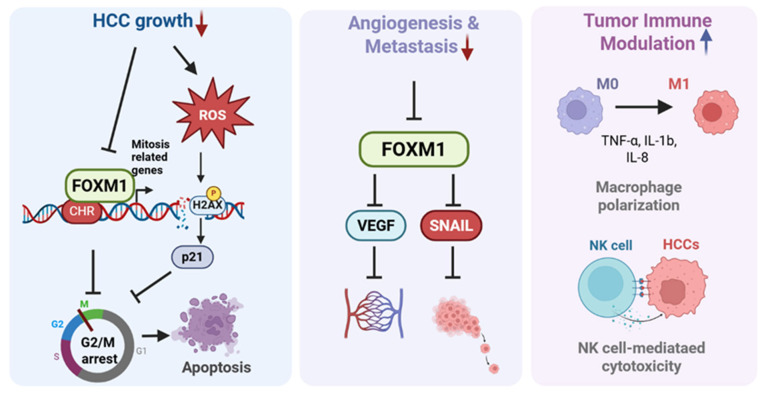
Multifaceted Antitumor Mechanism of HO-1197 in HCC. HO-1197 exhibits multifunctional antitumor effects, including inhibition of HCC growth, downregulation of angiogenesis, and metastasis, as well as modulation of tumor immune responses. It suppresses FoxM1 expression and promotes ROS accumulation, leading to G2/M cell cycle arrest and subsequent tumor cell death. In addition, HO-1197 attenuates angiogenesis and metastasis by downregulating FoxM1. It induces M1 macrophage polarization and NK cell-mediated cytotoxicity, resulting in tumor cell death.

In conclusion, HO-1197 exemplifies a new generation of evidence-based herbal therapeutics that target multiple hallmarks of HCC, including cell cycle deregulation, immune suppression, angiogenesis, and metastasis. Its ability to overcome p53 mutation, inhibit FOXM1-driven proliferation, and reshape the TME suggests that HO-1197 may fill critical gaps in current treatment paradigms [Figure 9].

## Data Availability

The original contributions presented in this study are included in the article/Supplementary Materials.

## Supplementary Materials

The following supporting information can be downloaded at: https://www.mdpi.com/article/10.3390/ijms262110329/s1.
