# Supplementary material for: HO-1197 as a Multifaceted Therapeutic: Targeting the Cell Cycle, Angiogenesis, Metastasis, and Tumor Immunity in Hepatocellular Carcinoma
Source: Int J Mol Sci. 2025 Oct 23;26(21):10329. doi: 10.3390/ijms262110329 (PMC12609843; doi:10.3390/ijms262110329)
Supplement: Supplementary file 1 [file ijms-26-10329-s001.zip › ijms-3856490-supplementary/Supplementary Table S1-1.pdf]

Supplementary Table S1. Antibody list

Antibody Name	Manufacturer	Catalog No.
CD133/1 (W6B3C1)	Miltenyi Biotec	130-092-395
PARP	Cell Signaling Technology	9532S
Caspase-3	Cell Signaling Technology	9662S
Cleaved caspase-3	Abcam	ab32042
γ-H2AX	Merck Millipore	05-636
p21	Cell Signaling Technology	2947S
p-p53(S15)	Cell Signaling Technology	9284S
FoxM1	Abcam	ab207298
Aurora A	Cell Signaling Technology	14475S
Bub 1	Abcam	ab195268
TTK/Msp1	Abcam	ab11108
PLK1	Cell Signaling Technology	4513S
CDC2	Abcam	ab183479
VEGF-A	Cell Signaling Technology	50661S
VEGFR2	Abcam	ab39256
Tie2	Cell Signaling Technology	4224S
N-cadherin	Abcam	ab76057
Vimentin	Abcam	ab8978
Snail	Cell Signaling Technology	3879S
α-SMA	Abcam	ab32575
β-actin	Sigma-aldrich	A5441
