# Supplementary figures and images for: HO-1197 as a Multifaceted Therapeutic: Targeting the Cell Cycle, Angiogenesis, Metastasis, and Tumor Immunity in Hepatocellular Carcinoma
Source: Int J Mol Sci. 2025 Oct 23;26(21):10329. doi: 10.3390/ijms262110329 (PMC12609843; doi:10.3390/ijms262110329)

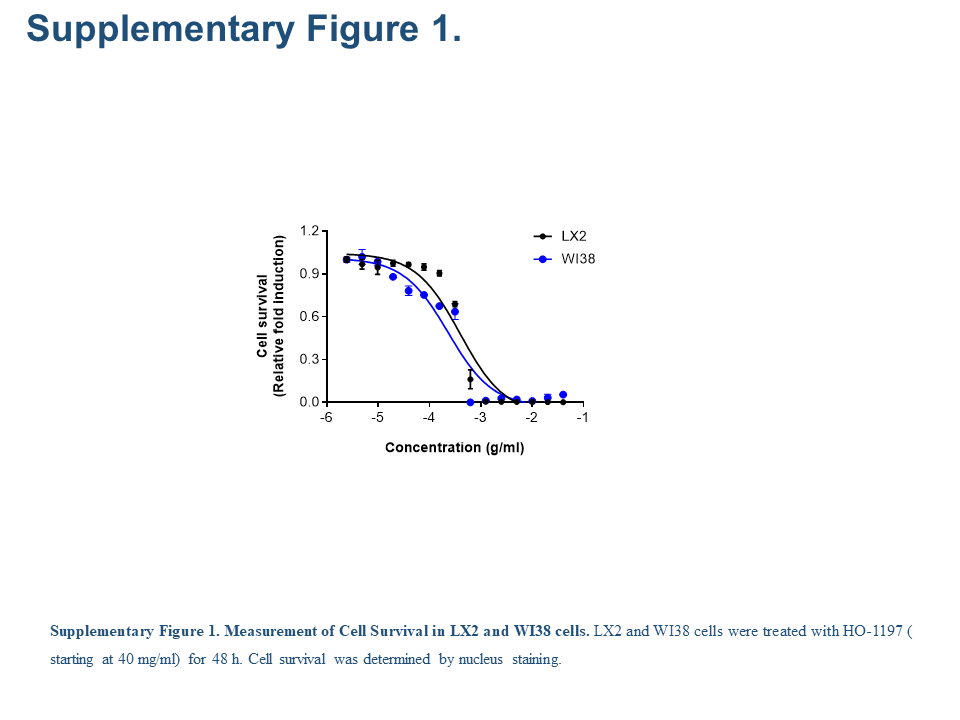

Supplement: Supplementary file 1 [file ijms-26-10329-s001.zip › ijms-3856490-supplementary/HO-1197_supple_revision_250926/Supplementary Figure 1.TIF]

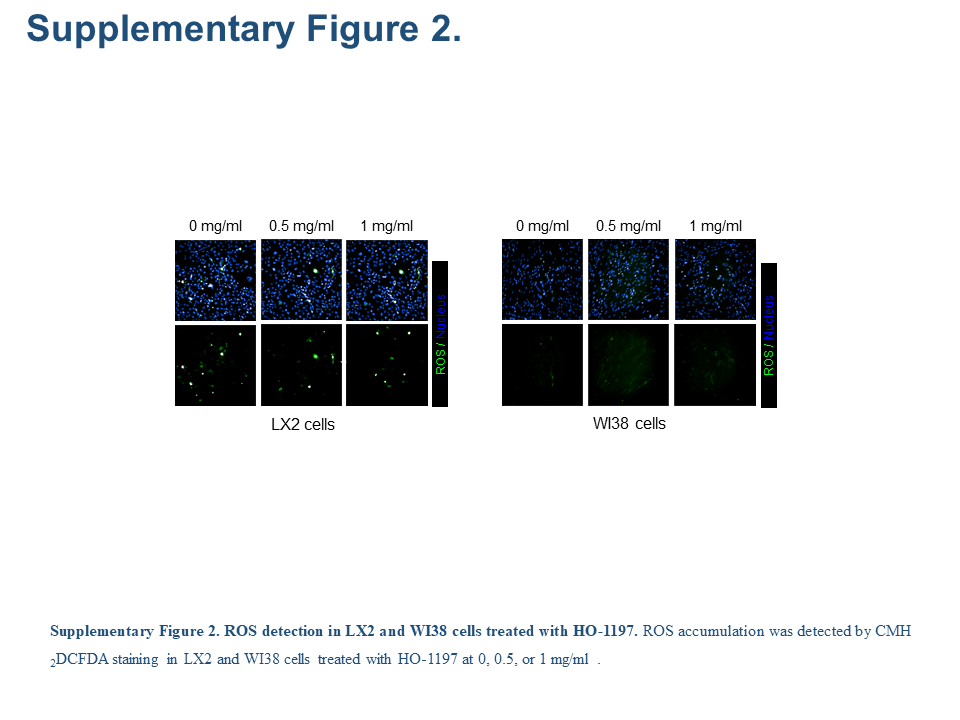

Supplement: Supplementary file 1 [file ijms-26-10329-s001.zip › ijms-3856490-supplementary/HO-1197_supple_revision_250926/Supplementary Figure 2.TIF]

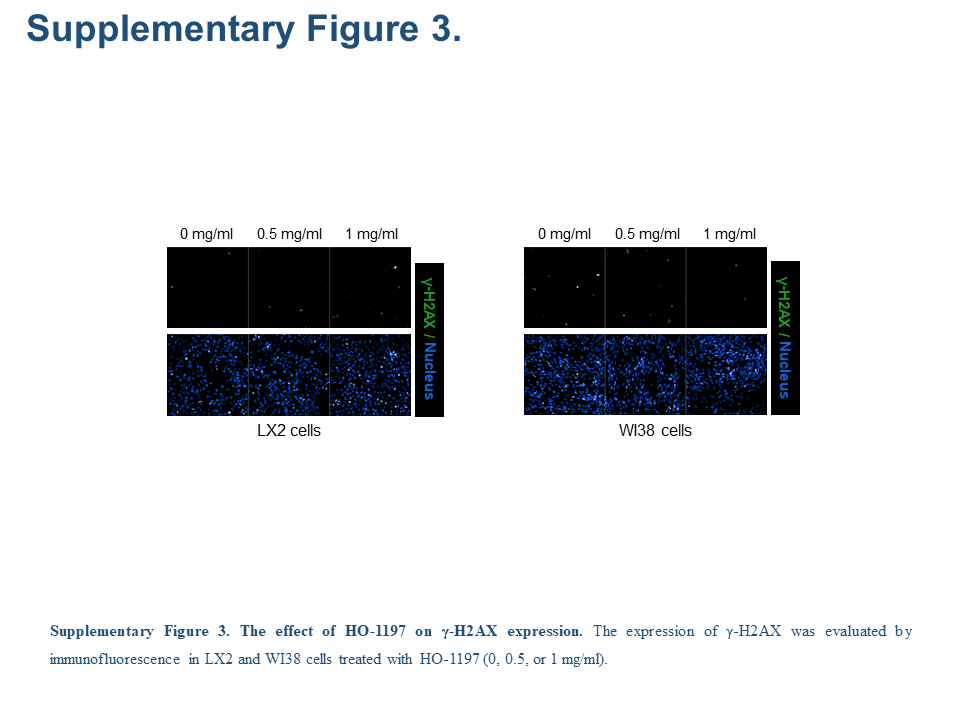

Supplement: Supplementary file 1 [file ijms-26-10329-s001.zip › ijms-3856490-supplementary/HO-1197_supple_revision_250926/Supplementary Figure 3.TIF]

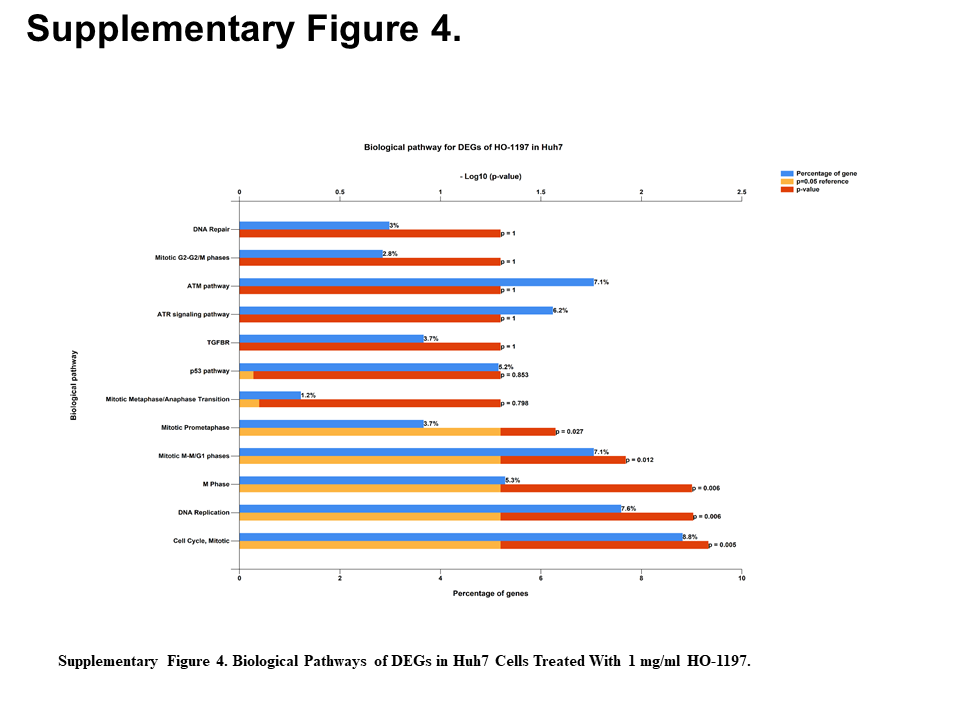

Supplement: Supplementary file 1 [file ijms-26-10329-s001.zip › ijms-3856490-supplementary/HO-1197_supple_revision_250926/Supplementary Figure 4.TIF]

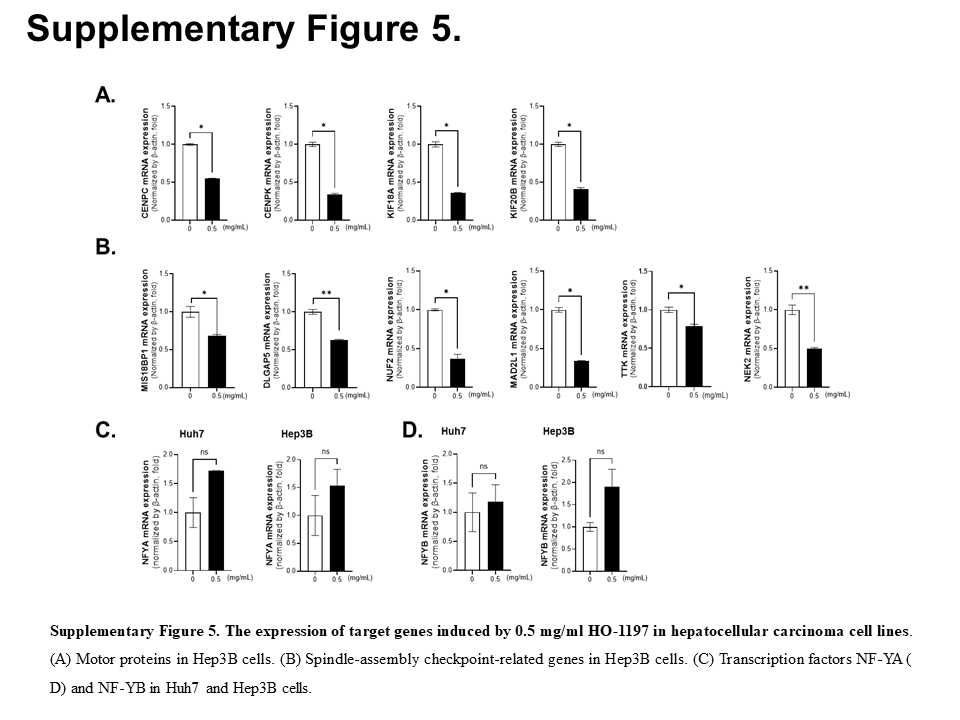

Supplement: Supplementary file 1 [file ijms-26-10329-s001.zip › ijms-3856490-supplementary/HO-1197_supple_revision_250926/Supplementary Figure 5.TIF]

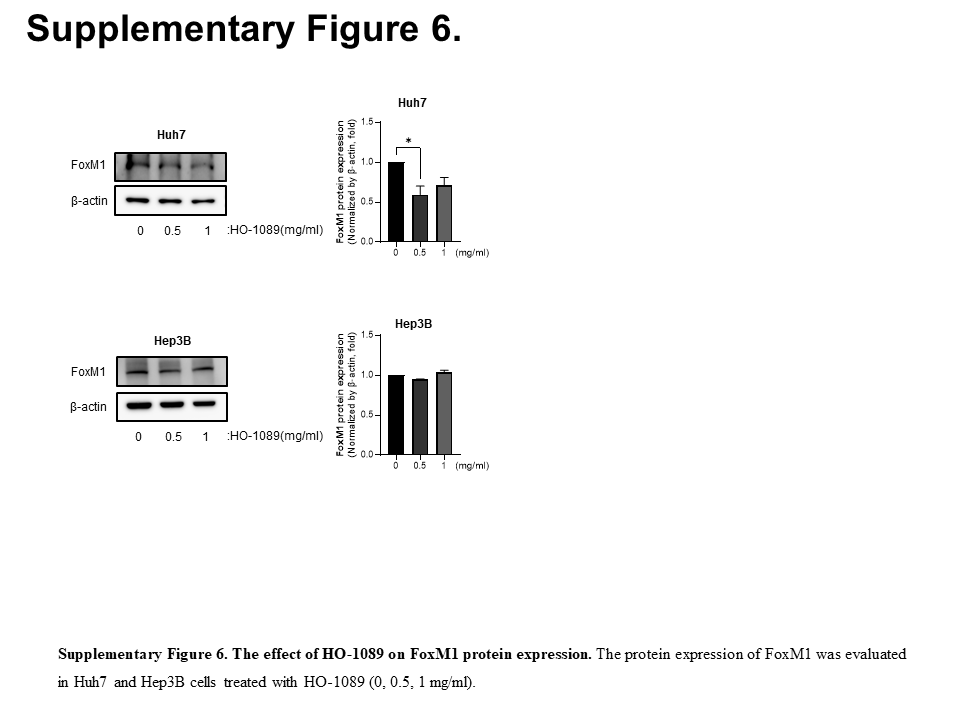

Supplement: Supplementary file 1 [file ijms-26-10329-s001.zip › ijms-3856490-supplementary/HO-1197_supple_revision_250926/Supplementary Figure 6.TIF]

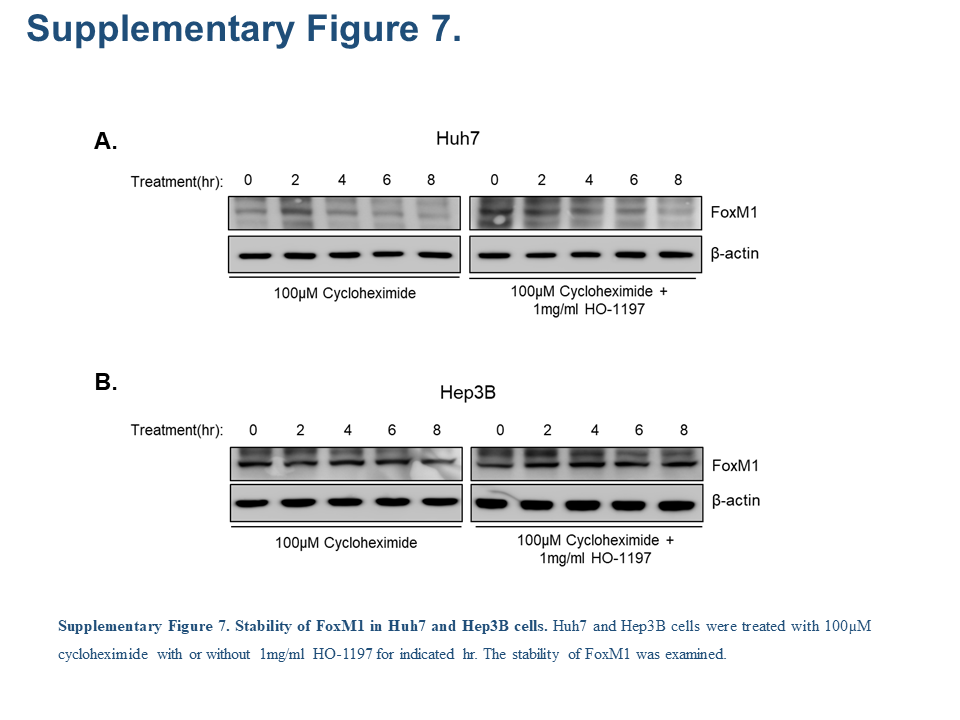

Supplement: Supplementary file 1 [file ijms-26-10329-s001.zip › ijms-3856490-supplementary/HO-1197_supple_revision_250926/Supplementary Figure 7.TIF]

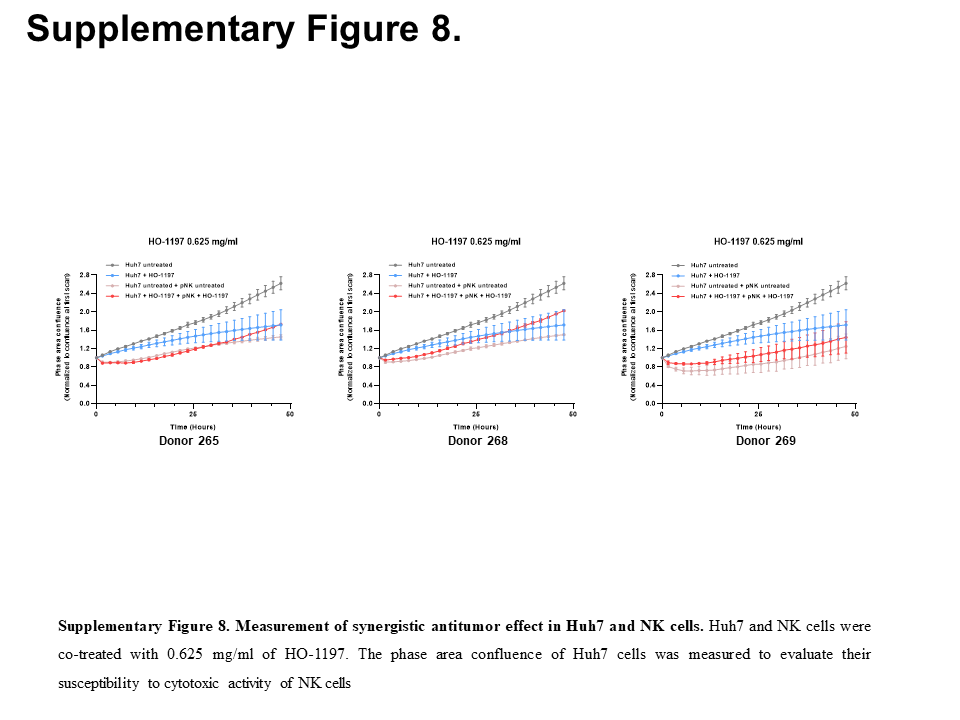

Supplement: Supplementary file 1 [file ijms-26-10329-s001.zip › ijms-3856490-supplementary/HO-1197_supple_revision_250926/Supplementary Figure 8.TIF]
